# 3-Methyl-4-[2-(4-nitro­phen­yl)hydrazin-1-yl­idene]-5-oxo-4,5-dihydro-1*H*-pyrazole-1-carbothio­amide

**DOI:** 10.1107/S1600536812027134

**Published:** 2012-06-23

**Authors:** Hoong-Kun Fun, Ching Kheng Quah, Shobhitha Shetty, Balakrishna Kalluraya, M. Babu

**Affiliations:** a X-ray Crystallography Unit, School of Physics, Universiti Sains Malaysia, 11800 USM, Penang, Malaysia; b Department of Studies in Chemistry, Mangalore University, Mangalagangotri, Mangalore 574 199, India

## Abstract

The asymmetric unit of the title compound, C_11_H_10_N_6_O_3_S, contains two independent mol­ecules, each of which is stabilized by an intra­molecular N—H⋯O hydrogen bond, forming an *S*(6) ring motif. In one mol­ecule, the pyrazole ring forms a dihedral angle of 10.93 (14)° with the benzene ring. The corresponding dihedral angle in the other mol­ecule is 7.03 (14)°. In the crystal, mol­ecules are linked *via* pairs of (N,N)—H⋯O bifurcated acceptor bonds which, together with C—H⋯O hydrogen bonds, form sheets parallel to (001).

## Related literature

For general background to and the pharmacological activity of pyrazole derivatives, see: Isloor *et al.* (2009 ▶); Rai *et al.* (2008 ▶); Bradbury & Pucci (2008 ▶); Girisha *et al.* (2010 ▶). For standard bond-length data, see: Allen *et al.* (1987 ▶). For the stability of the temperature controller used in the data collection, see Cosier & Glazer (1986 ▶). For hydrogen-bond motifs, see: Bernstein *et al.* (1995 ▶).
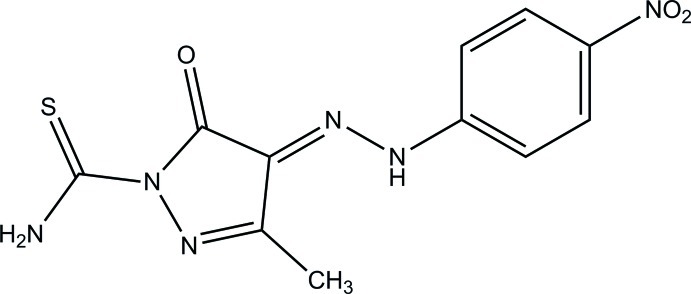


## Experimental

### 

#### Crystal data

C_11_H_10_N_6_O_3_S
*M* *_r_* = 306.31
Monoclinic, 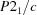

*a* = 11.5331 (4) Å
*b* = 17.2540 (6) Å
*c* = 13.6025 (5) Å
β = 105.840 (2)°
*V* = 2604.01 (16) Å^3^
*Z* = 8
Mo *K*α radiation
μ = 0.27 mm^−1^
*T* = 100 K
0.23 × 0.19 × 0.13 mm

#### Data collection

Bruker SMART APEXII CCD area-detector diffractometer
Absorption correction: multi-scan (*SADABS*; Bruker, 2009 ▶) *T*_min_ = 0.940, *T*_max_ = 0.965
29927 measured reflections
7706 independent reflections
5153 reflections with *I* > 2σ(*I*)
*R*_int_ = 0.086

#### Refinement

*R*[*F* ^2^ > 2σ(*F* ^2^)] = 0.074
*wR*(*F* ^2^) = 0.187
*S* = 1.05
7706 reflections
405 parameters
H atoms treated by a mixture of independent and constrained refinement
Δρ_max_ = 1.17 e Å^−3^
Δρ_min_ = −0.45 e Å^−3^

### 

Data collection: *APEX2* (Bruker, 2009 ▶); cell refinement: *SAINT* (Bruker, 2009 ▶); data reduction: *SAINT*; program(s) used to solve structure: *SHELXTL* (Sheldrick, 2008 ▶); program(s) used to refine structure: *SHELXTL*; molecular graphics: *SHELXTL*; software used to prepare material for publication: *SHELXTL* and *PLATON* (Spek, 2009 ▶).

## Supplementary Material

Crystal structure: contains datablock(s) global, I. DOI: 10.1107/S1600536812027134/kj2205sup1.cif

Structure factors: contains datablock(s) I. DOI: 10.1107/S1600536812027134/kj2205Isup2.hkl

Supplementary material file. DOI: 10.1107/S1600536812027134/kj2205Isup3.cml

Additional supplementary materials:  crystallographic information; 3D view; checkCIF report

## Figures and Tables

**Table 1:** Hydrogen-bond geometry (Å, °)

*D*—H⋯*A*	*D*—H	H⋯*A*	*D*⋯*A*	*D*—H⋯*A*
N5*B*—H2*N*5⋯O3*B*	0.97 (3)	2.08 (3)	2.810 (3)	131 (3)
N1*A*—H1*N*1⋯O3*B* ^i^	0.88 (4)	2.00 (4)	2.859 (3)	165 (4)
N5*A*—H1*N*5⋯O3*A*	0.92 (3)	2.11 (4)	2.802 (3)	131 (3)
N1*B*—H3*N*1⋯O3*A* ^ii^	0.87 (3)	1.99 (4)	2.848 (3)	171 (3)
C10*B*—H10*B*⋯O2*A* ^iii^	0.95	2.51	3.418 (3)	161

Symmetry codes: (i) 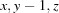; (ii) 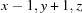; (iii) 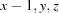.

**Fig. 1.**
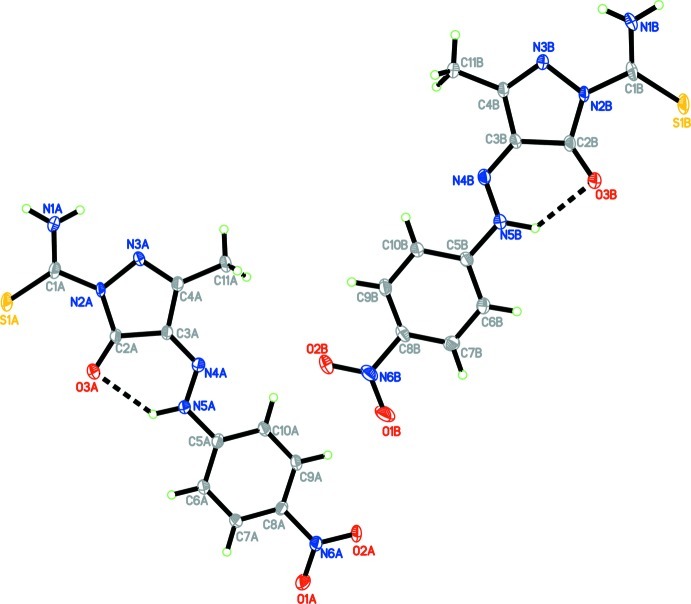
The molecular structure of the title compound showing 50% probability displacement ellipsoids for non-H atoms. Intramolecular bonds are shown as dashed lines.

**Fig. 2.**
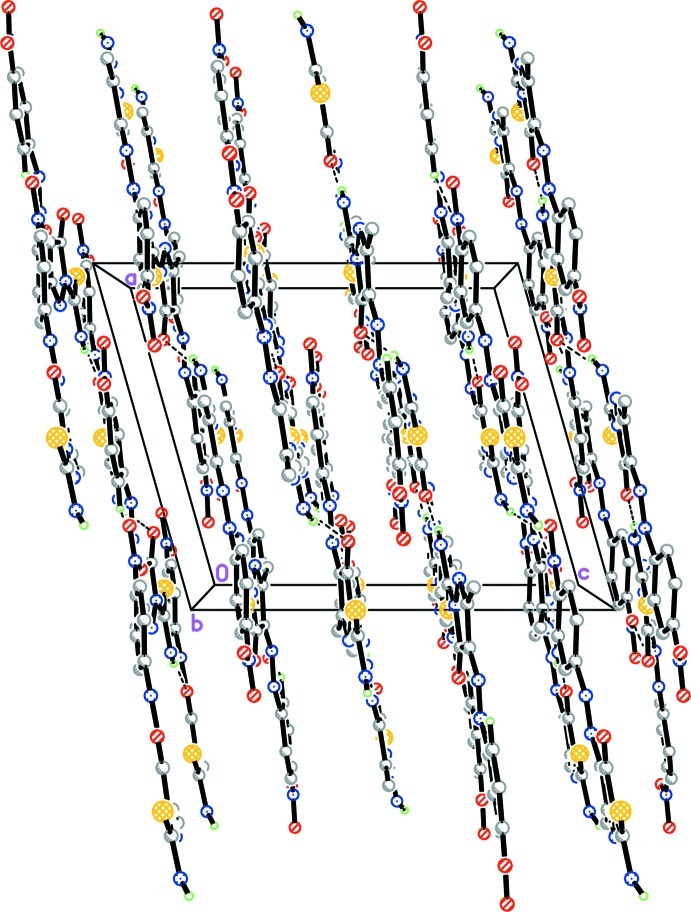
The crystal structure of the title compound, viewed along the b axis. H atoms not involved in hydrogen bonds (dashed lines) have been omitted for clarity.

## supplementary crystallographic information

### Comment

The pyrazole ring is a prominent structural moiety found in numerous pharmaceutically active compounds. This is mainly due to the easy preparation and the important pharmacological activity. Therefore, the synthesis and selective functionalization of pyrazoles have been the focus of active research over the years (Isloor *et al.*, 2009). Pyrazoles have been reported to possess antibacterial activity (Rai *et al.*, 2008), and inhibitor activity against DNA gyrase and topoisomerase IV at their respective ATP-binding sites (Bradbury & Pucci, 2008). Moreover, pyrazole-containing compounds have received considerable attention owing to their diverse chemotherapeutic potentials including versatile anti-inflammatory and antimicrobial activities (Girisha *et al.*, 2010). The synthetic route followed for obtaining the title compound involves the diazotization of substituted anilines to give the diazonium salts followed by coupling with ethyl acetoacetate in the presence of sodium acetate to give the corresponding oxobutanoate which on further reaction with thiosemicarbazide in acetic acid gave the required thioamides.

The asymmetric unit contains two independent molecules (Fig. 1), *A* and *B*. Each molecule is stabilized by an intramolecular N–H···O hydrogen bond (Table 1), forming a S(6) ring motif (Bernstein *et al.*, 1995). In molecule *A*, the pyrazole ring (N2A/N3A/C2A-C4A) forms a dihedral angle of 10.93 (14)° with the benzene ring (C5A-C10A). The corresponding dihedral angle in the molecule *B* is 7.03 (14)°. Bond lengths (Allen *et al.*, 1987) and angles are within normal ranges.

In the crystal (Fig.2), molecules are linked *via* pairs of intermolecular N5B–H2N5···O3B, N1A–H1N1···O3B and N5A–H1N5···O3A, N1B–H3N1···O3A bifurcated acceptor bonds (Table 1) which together with C10B–H10B···O2A hydrogen bonds form two-dimensional sheets parallel to (001).

### Experimental

To a solution of ethyl-2-[(4-nitrophenyl)hydrazono]-3-oxobutanoate (0.01 mol) dissolved in glacial acetic acid (20 ml), a solution of thiosemicarbazide (0.02 mol) in glacial acetic acid (25 ml) was added and the mixture was refluxed for 4 h. This was cooled and allowed to stand overnight. The solid product which separated out was filtered and dried. It was then recrystallized from ethanol. Crystals suitable for X-ray analysis were obtained by slow evaporation of a solution of the title compound in a 1:2 mixture of DMF and ethanol.

### Refinement

N-bound H atoms were located in a difference Fourier map and refined freely [N–H = 0.84 (4)- 0.98 (4) Å]. The rest of hydrogen atoms were positioned geometrically and refined using a riding model with C–H = 0.95 or 0.98 Å and *U*_iso_(H) = 1.2 or 1.5 *U*_eq_(C). A rotating-group model was applied for the methyl groups.

### Crystal data

**Table:**

C_11_H_10_N_6_O_3_S	*F*(000) = 1264
*M**_r_* = 306.31	*D*_x_ = 1.563 Mg m^−^^3^
Monoclinic, *P*2_1_/*c*	Mo *K*α radiation, λ = 0.71073 Å
Hall symbol: -P 2ybc	Cell parameters from 4616 reflections
*a* = 11.5331 (4) Å	θ = 2.4–29.9°
*b* = 17.2540 (6) Å	µ = 0.27 mm^−^^1^
*c* = 13.6025 (5) Å	*T* = 100 K
β = 105.840 (2)°	Block, orange
*V* = 2604.01 (16) Å^3^	0.23 × 0.19 × 0.13 mm
*Z* = 8	

### Data collection

**Table:**

Bruker SMART APEXII CCD area-detector diffractometer	7706 independent reflections
Radiation source: fine-focus sealed tube	5153 reflections with *I* > 2σ(*I*)
Graphite monochromator	*R*_int_ = 0.086
φ and ω scans	θ_max_ = 30.2°, θ_min_ = 2.4°
Absorption correction: multi-scan (*SADABS*; Bruker, 2009)	*h* = −16→14
*T*_min_ = 0.940, *T*_max_ = 0.965	*k* = −24→22
29927 measured reflections	*l* = −19→19

### Refinement

**Table:**

Refinement on *F*^2^	Primary atom site location: structure-invariant direct methods
Least-squares matrix: full	Secondary atom site location: difference Fourier map
*R*[*F*^2^ > 2σ(*F*^2^)] = 0.074	Hydrogen site location: inferred from neighbouring sites
*wR*(*F*^2^) = 0.187	H atoms treated by a mixture of independent and constrained refinement
*S* = 1.05	*w* = 1/[σ^2^(*F*_o_^2^) + (0.0947*P*)^2^ + 0.6575*P*] where *P* = (*F*_o_^2^ + 2*F*_c_^2^)/3
7706 reflections	(Δ/σ)_max_ = 0.001
405 parameters	Δρ_max_ = 1.17 e Å^−^^3^
0 restraints	Δρ_min_ = −0.45 e Å^−^^3^

### Special details

**Table:**

Experimental. The crystal was placed in the cold stream of an Oxford Cryosystems Cobra open-flow nitrogen cryostat (Cosier & Glazer, 1986) operating at 100.0 (1) K.
Geometry. All esds (except the esd in the dihedral angle between two l.s. planes) are estimated using the full covariance matrix. The cell esds are taken into account individually in the estimation of esds in distances, angles and torsion angles; correlations between esds in cell parameters are only used when they are defined by crystal symmetry. An approximate (isotropic) treatment of cell esds is used for estimating esds involving l.s. planes.
Refinement. Refinement of F^2^ against ALL reflections. The weighted R-factor wR and goodness of fit S are based on F^2^, conventional R-factors R are based on F, with F set to zero for negative F^2^. The threshold expression of F^2^ > 2sigma(F^2^) is used only for calculating R-factors(gt) etc. and is not relevant to the choice of reflections for refinement. R-factors based on F^2^ are statistically about twice as large as those based on F, and R- factors based on ALL data will be even larger.

### Fractional atomic coordinates and isotropic or equivalent isotropic displacement parameters (Å^2^)

**Table:**

	*x*	*y*	*z*	*U*_iso_*/*U*_eq_	
S1A	0.49627 (7)	0.07456 (4)	0.34900 (6)	0.02459 (18)	
O1A	1.23941 (18)	0.56130 (12)	0.40495 (19)	0.0319 (5)	
O2A	1.10770 (18)	0.65219 (11)	0.39347 (18)	0.0290 (5)	
O3A	0.67596 (17)	0.21463 (11)	0.36767 (15)	0.0218 (4)	
N1A	0.2988 (2)	0.15650 (15)	0.3363 (2)	0.0228 (5)	
N2A	0.46510 (19)	0.22947 (12)	0.34390 (17)	0.0167 (4)	
N3A	0.38600 (19)	0.29408 (12)	0.33564 (18)	0.0183 (5)	
N4A	0.65953 (19)	0.38832 (13)	0.35212 (16)	0.0171 (4)	
N5A	0.77001 (19)	0.36389 (13)	0.36300 (17)	0.0172 (4)	
N6A	1.1355 (2)	0.58362 (13)	0.39233 (18)	0.0193 (5)	
C1A	0.4155 (2)	0.15527 (14)	0.3430 (2)	0.0182 (5)	
C2A	0.5845 (2)	0.25291 (14)	0.35571 (19)	0.0154 (5)	
C3A	0.5750 (2)	0.33756 (14)	0.35069 (19)	0.0157 (5)	
C4A	0.4505 (2)	0.35624 (14)	0.3387 (2)	0.0176 (5)	
C5A	0.8602 (2)	0.41880 (14)	0.36467 (19)	0.0163 (5)	
C6A	0.9803 (2)	0.39484 (15)	0.3935 (2)	0.0179 (5)	
H6AA	0.9996	0.3418	0.4083	0.021*	
C7A	1.0711 (2)	0.44859 (15)	0.4003 (2)	0.0182 (5)	
H7AA	1.1532	0.4331	0.4194	0.022*	
C8A	1.0399 (2)	0.52539 (15)	0.37867 (19)	0.0165 (5)	
C9A	0.9203 (2)	0.55040 (15)	0.3490 (2)	0.0182 (5)	
H9AA	0.9015	0.6036	0.3349	0.022*	
C10A	0.8299 (2)	0.49643 (15)	0.3407 (2)	0.0182 (5)	
H10A	0.7478	0.5118	0.3189	0.022*	
C11A	0.3981 (3)	0.43569 (15)	0.3332 (2)	0.0253 (6)	
H11A	0.3103	0.4327	0.3057	0.038*	
H11B	0.4315	0.4681	0.2883	0.038*	
H11C	0.4181	0.4584	0.4017	0.038*	
S1B	0.01234 (7)	1.16479 (4)	0.38971 (6)	0.02669 (19)	
O1B	0.77974 (19)	0.69756 (14)	0.4551 (2)	0.0433 (7)	
O2B	0.65439 (19)	0.60220 (12)	0.43636 (18)	0.0322 (5)	
O3B	0.19810 (17)	1.02711 (11)	0.41542 (15)	0.0217 (4)	
N1B	−0.1838 (2)	1.07880 (16)	0.3673 (2)	0.0268 (6)	
N2B	−0.0135 (2)	1.00943 (12)	0.37488 (17)	0.0173 (4)	
N3B	−0.0931 (2)	0.94476 (12)	0.35002 (17)	0.0180 (5)	
N4B	0.1857 (2)	0.85527 (12)	0.37418 (16)	0.0170 (4)	
N5B	0.2965 (2)	0.88122 (13)	0.39365 (18)	0.0187 (5)	
N6B	0.6769 (2)	0.67177 (14)	0.43667 (19)	0.0240 (5)	
C1B	−0.0669 (3)	1.08329 (14)	0.3765 (2)	0.0202 (5)	
C2B	0.1063 (2)	0.98794 (14)	0.39107 (19)	0.0161 (5)	
C3B	0.0986 (2)	0.90382 (14)	0.3735 (2)	0.0163 (5)	
C4B	−0.0271 (2)	0.88367 (14)	0.3494 (2)	0.0164 (5)	
C5B	0.3899 (2)	0.82832 (15)	0.39719 (19)	0.0166 (5)	
C6B	0.5065 (2)	0.85661 (15)	0.4119 (2)	0.0198 (5)	
H6BA	0.5207	0.9109	0.4151	0.024*	
C7B	0.6014 (2)	0.80571 (16)	0.4220 (2)	0.0204 (5)	
H7BA	0.6814	0.8242	0.4319	0.024*	
C8B	0.5766 (2)	0.72671 (15)	0.4171 (2)	0.0195 (5)	
C9B	0.4603 (2)	0.69750 (15)	0.3996 (2)	0.0206 (5)	
H9BA	0.4462	0.6432	0.3947	0.025*	
C10B	0.3651 (2)	0.74874 (14)	0.3893 (2)	0.0184 (5)	
H10B	0.2849	0.7303	0.3772	0.022*	
C11B	−0.0786 (2)	0.80485 (15)	0.3263 (2)	0.0227 (6)	
H11D	−0.1666	0.8083	0.3017	0.034*	
H11E	−0.0561	0.7731	0.3884	0.034*	
H11F	−0.0470	0.7810	0.2735	0.034*	
H2N1	0.263 (3)	0.200 (2)	0.335 (3)	0.044 (11)*	
H2N5	0.313 (3)	0.936 (2)	0.407 (2)	0.032 (9)*	
H1N1	0.261 (3)	0.115 (2)	0.349 (3)	0.042 (11)*	
H4N1	−0.215 (4)	1.035 (3)	0.359 (3)	0.049 (12)*	
H1N5	0.788 (3)	0.312 (2)	0.370 (3)	0.033 (9)*	
H3N1	−0.223 (3)	1.122 (2)	0.362 (3)	0.038 (10)*	

### Atomic displacement parameters (Å^2^)

**Table:**

	*U*^11^	*U*^22^	*U*^33^	*U*^12^	*U*^13^	*U*^23^
S1A	0.0290 (4)	0.0084 (3)	0.0381 (4)	−0.0001 (3)	0.0121 (3)	−0.0029 (3)
O1A	0.0179 (10)	0.0187 (11)	0.0617 (15)	−0.0028 (8)	0.0156 (10)	−0.0040 (10)
O2A	0.0235 (10)	0.0090 (9)	0.0543 (14)	−0.0009 (8)	0.0102 (10)	0.0037 (8)
O3A	0.0193 (9)	0.0125 (9)	0.0339 (11)	0.0033 (7)	0.0080 (8)	−0.0001 (7)
N1A	0.0193 (12)	0.0125 (11)	0.0360 (14)	−0.0033 (9)	0.0063 (10)	0.0034 (10)
N2A	0.0182 (10)	0.0062 (9)	0.0275 (12)	0.0016 (8)	0.0091 (9)	0.0004 (8)
N3A	0.0162 (10)	0.0107 (10)	0.0285 (12)	0.0029 (8)	0.0069 (9)	0.0023 (8)
N4A	0.0166 (10)	0.0135 (10)	0.0218 (11)	−0.0006 (8)	0.0061 (8)	−0.0002 (8)
N5A	0.0146 (10)	0.0117 (10)	0.0259 (12)	0.0006 (8)	0.0063 (9)	0.0000 (8)
N6A	0.0181 (11)	0.0132 (10)	0.0281 (12)	−0.0011 (8)	0.0089 (9)	0.0009 (8)
C1A	0.0228 (13)	0.0106 (11)	0.0207 (13)	−0.0027 (10)	0.0050 (10)	−0.0018 (9)
C2A	0.0168 (12)	0.0111 (11)	0.0203 (12)	−0.0003 (9)	0.0081 (9)	0.0003 (9)
C3A	0.0170 (12)	0.0106 (11)	0.0204 (12)	−0.0004 (9)	0.0068 (9)	0.0009 (9)
C4A	0.0186 (12)	0.0114 (12)	0.0244 (13)	−0.0001 (9)	0.0088 (10)	0.0009 (9)
C5A	0.0179 (12)	0.0126 (12)	0.0203 (13)	−0.0018 (9)	0.0083 (10)	−0.0014 (9)
C6A	0.0187 (12)	0.0127 (12)	0.0224 (13)	0.0014 (10)	0.0060 (10)	0.0014 (9)
C7A	0.0147 (12)	0.0146 (12)	0.0259 (14)	0.0022 (9)	0.0065 (10)	−0.0006 (10)
C8A	0.0166 (12)	0.0130 (12)	0.0211 (13)	−0.0029 (9)	0.0074 (10)	−0.0022 (9)
C9A	0.0204 (12)	0.0110 (11)	0.0235 (13)	0.0023 (9)	0.0066 (10)	0.0004 (9)
C10A	0.0183 (12)	0.0135 (12)	0.0239 (13)	0.0027 (9)	0.0076 (10)	0.0012 (9)
C11A	0.0252 (14)	0.0117 (12)	0.0409 (17)	0.0033 (11)	0.0124 (12)	0.0063 (11)
S1B	0.0341 (4)	0.0078 (3)	0.0424 (4)	0.0005 (3)	0.0175 (3)	0.0010 (3)
O1B	0.0184 (10)	0.0305 (13)	0.084 (2)	0.0083 (9)	0.0201 (11)	0.0189 (12)
O2B	0.0283 (11)	0.0157 (10)	0.0526 (14)	0.0077 (9)	0.0109 (10)	−0.0033 (9)
O3B	0.0201 (9)	0.0125 (9)	0.0339 (11)	−0.0004 (7)	0.0098 (8)	0.0006 (7)
N1B	0.0234 (12)	0.0125 (12)	0.0452 (16)	0.0068 (10)	0.0108 (11)	−0.0016 (10)
N2B	0.0198 (11)	0.0070 (9)	0.0268 (12)	−0.0001 (8)	0.0095 (9)	0.0000 (8)
N3B	0.0179 (10)	0.0099 (10)	0.0269 (12)	−0.0006 (8)	0.0074 (9)	0.0000 (8)
N4B	0.0198 (11)	0.0116 (10)	0.0204 (11)	0.0015 (8)	0.0067 (9)	0.0020 (8)
N5B	0.0173 (10)	0.0124 (10)	0.0266 (12)	0.0007 (8)	0.0061 (9)	−0.0008 (8)
N6B	0.0201 (11)	0.0223 (13)	0.0327 (13)	0.0078 (10)	0.0123 (10)	0.0054 (10)
C1B	0.0275 (14)	0.0096 (11)	0.0245 (14)	0.0057 (10)	0.0087 (11)	0.0021 (9)
C2B	0.0197 (12)	0.0091 (11)	0.0211 (13)	0.0008 (9)	0.0081 (10)	0.0035 (9)
C3B	0.0168 (12)	0.0098 (11)	0.0235 (13)	0.0019 (9)	0.0074 (10)	0.0010 (9)
C4B	0.0192 (12)	0.0093 (11)	0.0214 (12)	0.0007 (9)	0.0069 (10)	0.0001 (9)
C5B	0.0173 (12)	0.0143 (12)	0.0186 (12)	0.0024 (9)	0.0057 (9)	0.0006 (9)
C6B	0.0210 (13)	0.0135 (12)	0.0250 (13)	−0.0005 (10)	0.0061 (10)	0.0034 (10)
C7B	0.0177 (12)	0.0206 (13)	0.0238 (13)	−0.0009 (10)	0.0075 (10)	0.0050 (10)
C8B	0.0216 (13)	0.0163 (13)	0.0226 (13)	0.0070 (10)	0.0095 (10)	0.0022 (10)
C9B	0.0243 (13)	0.0130 (12)	0.0257 (14)	0.0021 (10)	0.0088 (11)	−0.0008 (10)
C10B	0.0177 (12)	0.0139 (12)	0.0244 (13)	0.0010 (10)	0.0074 (10)	0.0015 (10)
C11B	0.0202 (13)	0.0116 (12)	0.0366 (16)	−0.0009 (10)	0.0083 (11)	−0.0023 (10)

### Geometric parameters (Å, º)

**Table:**

S1A—C1A	1.665 (3)	S1B—C1B	1.660 (3)
O1A—N6A	1.226 (3)	O1B—N6B	1.227 (3)
O2A—N6A	1.227 (3)	O2B—N6B	1.228 (3)
O3A—C2A	1.218 (3)	O3B—C2B	1.223 (3)
N1A—C1A	1.324 (4)	N1B—C1B	1.322 (4)
N1A—H2N1	0.85 (4)	N1B—H4N1	0.84 (4)
N1A—H1N1	0.88 (4)	N1B—H3N1	0.86 (4)
N2A—C1A	1.401 (3)	N2B—C2B	1.389 (3)
N2A—C2A	1.401 (3)	N2B—C1B	1.418 (3)
N2A—N3A	1.425 (3)	N2B—N3B	1.426 (3)
N3A—C4A	1.299 (3)	N3B—C4B	1.302 (3)
N4A—C3A	1.306 (3)	N4B—C3B	1.305 (3)
N4A—N5A	1.312 (3)	N4B—N5B	1.312 (3)
N5A—C5A	1.403 (3)	N5B—C5B	1.403 (3)
N5A—H1N5	0.92 (4)	N5B—H2N5	0.98 (4)
N6A—C8A	1.465 (3)	N6B—C8B	1.463 (3)
C2A—C3A	1.465 (3)	C2B—C3B	1.470 (3)
C3A—C4A	1.438 (4)	C3B—C4B	1.439 (3)
C4A—C11A	1.492 (4)	C4B—C11B	1.483 (3)
C5A—C6A	1.395 (4)	C5B—C6B	1.392 (4)
C5A—C10A	1.400 (4)	C5B—C10B	1.401 (4)
C6A—C7A	1.383 (4)	C6B—C7B	1.381 (4)
C6A—H6AA	0.9500	C6B—H6BA	0.9500
C7A—C8A	1.383 (4)	C7B—C8B	1.391 (4)
C7A—H7AA	0.9500	C7B—H7BA	0.9500
C8A—C9A	1.396 (4)	C8B—C9B	1.392 (4)
C9A—C10A	1.379 (4)	C9B—C10B	1.386 (4)
C9A—H9AA	0.9500	C9B—H9BA	0.9500
C10A—H10A	0.9500	C10B—H10B	0.9500
C11A—H11A	0.9800	C11B—H11D	0.9800
C11A—H11B	0.9800	C11B—H11E	0.9800
C11A—H11C	0.9800	C11B—H11F	0.9800
			
C1A—N1A—H2N1	119 (3)	C1B—N1B—H4N1	117 (3)
C1A—N1A—H1N1	122 (3)	C1B—N1B—H3N1	117 (2)
H2N1—N1A—H1N1	117 (4)	H4N1—N1B—H3N1	125 (4)
C1A—N2A—C2A	130.6 (2)	C2B—N2B—C1B	130.9 (2)
C1A—N2A—N3A	117.6 (2)	C2B—N2B—N3B	112.16 (19)
C2A—N2A—N3A	111.77 (19)	C1B—N2B—N3B	116.9 (2)
C4A—N3A—N2A	107.1 (2)	C4B—N3B—N2B	107.2 (2)
C3A—N4A—N5A	118.9 (2)	C3B—N4B—N5B	119.2 (2)
N4A—N5A—C5A	118.6 (2)	N4B—N5B—C5B	118.8 (2)
N4A—N5A—H1N5	121 (2)	N4B—N5B—H2N5	120 (2)
C5A—N5A—H1N5	121 (2)	C5B—N5B—H2N5	121 (2)
O1A—N6A—O2A	123.4 (2)	O1B—N6B—O2B	123.2 (2)
O1A—N6A—C8A	118.4 (2)	O1B—N6B—C8B	118.3 (2)
O2A—N6A—C8A	118.2 (2)	O2B—N6B—C8B	118.5 (2)
N1A—C1A—N2A	113.0 (2)	N1B—C1B—N2B	112.5 (2)
N1A—C1A—S1A	124.1 (2)	N1B—C1B—S1B	125.2 (2)
N2A—C1A—S1A	122.8 (2)	N2B—C1B—S1B	122.3 (2)
O3A—C2A—N2A	130.3 (2)	O3B—C2B—N2B	130.2 (2)
O3A—C2A—C3A	126.7 (2)	O3B—C2B—C3B	126.8 (2)
N2A—C2A—C3A	103.0 (2)	N2B—C2B—C3B	103.0 (2)
N4A—C3A—C4A	124.7 (2)	N4B—C3B—C4B	124.9 (2)
N4A—C3A—C2A	128.5 (2)	N4B—C3B—C2B	128.3 (2)
C4A—C3A—C2A	106.7 (2)	C4B—C3B—C2B	106.7 (2)
N3A—C4A—C3A	111.3 (2)	N3B—C4B—C3B	111.0 (2)
N3A—C4A—C11A	122.5 (2)	N3B—C4B—C11B	122.9 (2)
C3A—C4A—C11A	126.2 (2)	C3B—C4B—C11B	126.1 (2)
C6A—C5A—C10A	121.0 (2)	C6B—C5B—C10B	121.6 (2)
C6A—C5A—N5A	118.6 (2)	C6B—C5B—N5B	118.6 (2)
C10A—C5A—N5A	120.4 (2)	C10B—C5B—N5B	119.8 (2)
C7A—C6A—C5A	119.7 (2)	C7B—C6B—C5B	120.0 (2)
C7A—C6A—H6AA	120.1	C7B—C6B—H6BA	120.0
C5A—C6A—H6AA	120.1	C5B—C6B—H6BA	120.0
C6A—C7A—C8A	118.7 (2)	C6B—C7B—C8B	118.1 (2)
C6A—C7A—H7AA	120.7	C6B—C7B—H7BA	121.0
C8A—C7A—H7AA	120.7	C8B—C7B—H7BA	121.0
C7A—C8A—C9A	122.5 (2)	C7B—C8B—C9B	122.6 (2)
C7A—C8A—N6A	119.1 (2)	C7B—C8B—N6B	119.0 (2)
C9A—C8A—N6A	118.3 (2)	C9B—C8B—N6B	118.3 (2)
C10A—C9A—C8A	118.7 (2)	C10B—C9B—C8B	119.1 (2)
C10A—C9A—H9AA	120.7	C10B—C9B—H9BA	120.5
C8A—C9A—H9AA	120.7	C8B—C9B—H9BA	120.5
C9A—C10A—C5A	119.5 (2)	C9B—C10B—C5B	118.6 (2)
C9A—C10A—H10A	120.3	C9B—C10B—H10B	120.7
C5A—C10A—H10A	120.3	C5B—C10B—H10B	120.7
C4A—C11A—H11A	109.5	C4B—C11B—H11D	109.5
C4A—C11A—H11B	109.5	C4B—C11B—H11E	109.5
H11A—C11A—H11B	109.5	H11D—C11B—H11E	109.5
C4A—C11A—H11C	109.5	C4B—C11B—H11F	109.5
H11A—C11A—H11C	109.5	H11D—C11B—H11F	109.5
H11B—C11A—H11C	109.5	H11E—C11B—H11F	109.5
			
C1A—N2A—N3A—C4A	179.6 (2)	C2B—N2B—N3B—C4B	−0.5 (3)
C2A—N2A—N3A—C4A	1.9 (3)	C1B—N2B—N3B—C4B	177.9 (2)
C3A—N4A—N5A—C5A	−179.9 (2)	C3B—N4B—N5B—C5B	178.2 (2)
C2A—N2A—C1A—N1A	176.0 (3)	C2B—N2B—C1B—N1B	−173.6 (3)
N3A—N2A—C1A—N1A	−1.2 (3)	N3B—N2B—C1B—N1B	8.4 (3)
C2A—N2A—C1A—S1A	−4.4 (4)	C2B—N2B—C1B—S1B	5.8 (4)
N3A—N2A—C1A—S1A	178.36 (18)	N3B—N2B—C1B—S1B	−172.19 (18)
C1A—N2A—C2A—O3A	0.5 (5)	C1B—N2B—C2B—O3B	3.6 (5)
N3A—N2A—C2A—O3A	177.8 (3)	N3B—N2B—C2B—O3B	−178.3 (3)
C1A—N2A—C2A—C3A	−179.3 (3)	C1B—N2B—C2B—C3B	−177.4 (3)
N3A—N2A—C2A—C3A	−2.0 (3)	N3B—N2B—C2B—C3B	0.7 (3)
N5A—N4A—C3A—C4A	−179.6 (2)	N5B—N4B—C3B—C4B	177.4 (2)
N5A—N4A—C3A—C2A	−2.7 (4)	N5B—N4B—C3B—C2B	0.7 (4)
O3A—C2A—C3A—N4A	4.2 (5)	O3B—C2B—C3B—N4B	−4.4 (5)
N2A—C2A—C3A—N4A	−176.0 (3)	N2B—C2B—C3B—N4B	176.5 (3)
O3A—C2A—C3A—C4A	−178.5 (3)	O3B—C2B—C3B—C4B	178.4 (3)
N2A—C2A—C3A—C4A	1.3 (3)	N2B—C2B—C3B—C4B	−0.7 (3)
N2A—N3A—C4A—C3A	−0.9 (3)	N2B—N3B—C4B—C3B	0.0 (3)
N2A—N3A—C4A—C11A	−179.3 (2)	N2B—N3B—C4B—C11B	−179.9 (2)
N4A—C3A—C4A—N3A	177.2 (2)	N4B—C3B—C4B—N3B	−176.9 (3)
C2A—C3A—C4A—N3A	−0.2 (3)	C2B—C3B—C4B—N3B	0.4 (3)
N4A—C3A—C4A—C11A	−4.5 (4)	N4B—C3B—C4B—C11B	3.0 (4)
C2A—C3A—C4A—C11A	178.1 (3)	C2B—C3B—C4B—C11B	−179.7 (3)
N4A—N5A—C5A—C6A	169.2 (2)	N4B—N5B—C5B—C6B	176.3 (2)
N4A—N5A—C5A—C10A	−9.0 (4)	N4B—N5B—C5B—C10B	−5.8 (4)
C10A—C5A—C6A—C7A	1.1 (4)	C10B—C5B—C6B—C7B	−1.8 (4)
N5A—C5A—C6A—C7A	−177.0 (2)	N5B—C5B—C6B—C7B	176.1 (2)
C5A—C6A—C7A—C8A	0.4 (4)	C5B—C6B—C7B—C8B	−0.1 (4)
C6A—C7A—C8A—C9A	−0.8 (4)	C6B—C7B—C8B—C9B	2.0 (4)
C6A—C7A—C8A—N6A	175.9 (2)	C6B—C7B—C8B—N6B	−174.6 (2)
O1A—N6A—C8A—C7A	12.7 (4)	O1B—N6B—C8B—C7B	−1.2 (4)
O2A—N6A—C8A—C7A	−165.3 (3)	O2B—N6B—C8B—C7B	177.0 (3)
O1A—N6A—C8A—C9A	−170.4 (3)	O1B—N6B—C8B—C9B	−177.9 (3)
O2A—N6A—C8A—C9A	11.6 (4)	O2B—N6B—C8B—C9B	0.3 (4)
C7A—C8A—C9A—C10A	−0.2 (4)	C7B—C8B—C9B—C10B	−1.9 (4)
N6A—C8A—C9A—C10A	−177.0 (2)	N6B—C8B—C9B—C10B	174.7 (2)
C8A—C9A—C10A—C5A	1.7 (4)	C8B—C9B—C10B—C5B	−0.1 (4)
C6A—C5A—C10A—C9A	−2.2 (4)	C6B—C5B—C10B—C9B	1.9 (4)
N5A—C5A—C10A—C9A	175.9 (2)	N5B—C5B—C10B—C9B	−175.9 (2)

### Hydrogen-bond geometry (Å, º)

**Table:**

*D*—H···*A*	*D*—H	H···*A*	*D*···*A*	*D*—H···*A*
N5*B*—H2*N*5···O3*B*	0.97 (3)	2.08 (3)	2.810 (3)	131 (3)
N1*A*—H1*N*1···O3*B*^i^	0.88 (4)	2.00 (4)	2.859 (3)	165 (4)
N5*A*—H1*N*5···O3*A*	0.92 (3)	2.11 (4)	2.802 (3)	131 (3)
N1*B*—H3*N*1···O3*A*^ii^	0.87 (3)	1.99 (4)	2.848 (3)	171 (3)
C10*B*—H10*B*···O2*A*^iii^	0.95	2.51	3.418 (3)	161

Symmetry codes: (i) *x*, *y*−1, *z*; (ii) *x*−1, *y*+1, *z*; (iii) *x*−1, *y*, *z*.
